# *Slc7a14* Is Indispensable in Zebrafish Retinas

**DOI:** 10.3389/fcell.2019.00333

**Published:** 2019-12-12

**Authors:** You-Yuan Zhuang, Lue Xiang, Xin-Ran Wen, Ren-Juan Shen, Ning Zhao, Si-Si Zheng, Ru-Yi Han, Jia Qu, Fan Lu, Zi-Bing Jin

**Affiliations:** ^1^Division of Ophthalmic Genetics, The Eye Hospital, Wenzhou Medical University, Wenzhou, China; ^2^State Key Laboratory of Ophthalmology, Optometry and Visual Science, National Clinical Research Center for Ophthalmology, National Center for International Research in Regenerative Medicine and Neurogenetics, Wenzhou, China

**Keywords:** *slc7a14*, retinitis pigmentosa, zebrafish, rod photoreceptors, RPE

## Abstract

Previous study has identified *SLC7A14* as a new causative gene of retinitis pigmentosa (RP). However, the role of *SLC7A14* has not been fully characterized. The goal of this study was to investigate the biological features of *slc7a14* in zebrafish. To determine the expression of *slc7a14* in developing zebrafish, we performed *in situ* hybridization (ISH) and quantitative real-time PCR. Morpholino knockdown and overexpression experiments were performed to study the role of *slc7a14* in zebrafish retinas. Immunostaining was carried out to observe structural changes. Visual motor responses (VMR) and optokinetic responses (OKR) were analyzed to assess visual behaviors. Terminal deoxynucleotidyl transferase (dUTP) nick-end labeling (TUNEL) staining was performed to survey apoptotic retinal cells. We found that *slc7a14* was highly expressed in neuronal tissues, including the brain, spinal cord and retina, and that the expression levels increased during early embryogenesis. Consistently, ISH showed a similar expression pattern. Knockdown of *slc7a14* led to dose-dependent microphthalmia that was reversed by overexpression. The immunostaining results revealed that the rod-specific protein zpr-3 and the retinal pigment epithelium-specific protein zpr-2 (decreased to 44.48%) were significantly suppressed in the *slc7a14*-silenced morphants. Notably, visual behaviors (the VMR and the OKR) were severely impaired in the *slc7a14*-deficient morphant, especially the VMR OFF response. In addition, apoptotic cells were observed in the retina at 3 days post fertilization (dpf) and 5 dpf by TUNEL assay. Our results demonstrated that *slc7a14* is essential for visually mediated behaviors in zebrafish. Temporary silencing of *slc7a14* in larvae led to severe visual impairments, consistent with the manifestations observed in RP patients. Our findings provide further insights into the genetic mechanisms of RP predisposition caused by *SLC7A14* mutations.

## Introduction

Retinitis pigmentosa (RP) is one of the leading causes of inherited middle-aged blindness worldwide. The disease is characterized by progressive rod photoreceptor death (Berson, 1993; Daiger et al., 2014) and subsequent cone loss (Hartong et al., 2006; Roche et al., 2019). To date, more than 4600 mutations in 89 genes have been discovered in RP (RetNet^1^ and RetinoGenetics^2^) (Daiger et al., 1998; Ran et al., 2014). Among the disease-causing genes of autosomal recessive RP (arRP), *SLC7A14* accounts for 2.02% in sporadic and recessive RP patients in China (Jin et al., 2014).

The *SLC7A14* gene (encoding solute carrier family 7 member 14) consists of 7 exons, and there is high sequence homology between the zebrafish and human genes (Jin et al., 2014). *SLC7A14* is considered a lysosomal transporter for cationic amino acids (Jaenecke et al., 2012). Based on gene ontology (GO) annotation, the function of *SLC7A14* was predicted as a transmembrane transporter for L-amino acid. Patients with *SLC7A14* mutations showed impaired vision, intraretinal bone spicule pigmentation, extinguished electroretinogram (ERG) responses and thinned outer retinal layers (Jin et al., 2014). *Slc7a14* knockout mice also display reduced ERG responses (Jin et al., 2014). However, the mechanisms by which *SLC7A14* mutations cause arRP have not been fully elucidated.

Zebrafish are ideal animal models for human retinal diseases due to their easier genetic manipulation, easier real-time observation (Raghupathy et al., 2013; Chhetri et al., 2014), and higher fecundity of zebrafish than of mice as well as the similar retinal anatomy between zebrafish and humans (Link and Collery, 2015; Zheng et al., 2018). To further characterize *slc7a14* in the zebrafish retina, we constructed an *slc7a14*-deficient model using morpholino oligonucleotide (MO)-induced knockdown. Temporary knockdown of *slc7a14* led to significant retinal degeneration, which was reversed by forced overexpression. Our results suggest that *slc7a14* is indispensable for retinal structure and function in zebrafish.

## Materials and Methods

### Zebrafish Husbandry and Embryo Preparation

Adult zebrafish of the Tg(gad1b:mCherry) strain were obtained from the China Zebrafish Resource Center. To generate transgenic constructs, a 2.3-kb sequence upstream of the zebrafish gad1b gene coding region was cloned and integrated with the mCherry gene (Song et al., 2017). The mCherry fluorescence in Tg(gad1b:mCherry) could be detected in the brain, olfactory pit, optic tectum, spinal cord as well as eye. In this study, we used the AB wild-type strain and the Tg(gad1b:mCherry) strain. The husbandry, breeding, embryo collection and incubation were performed according to standard procedures (Westerfield, 2000). All experiments were carried out in accordance with the Association for Research on Vision and Ophthalmology’s statement on the Use of Animals in Ophthalmic and Vision Research and were approved by the Institutional Animal Care and Use Committee of Wenzhou Medical University.

### Quantitative Real-Time PCR

**FIGURE 1:**
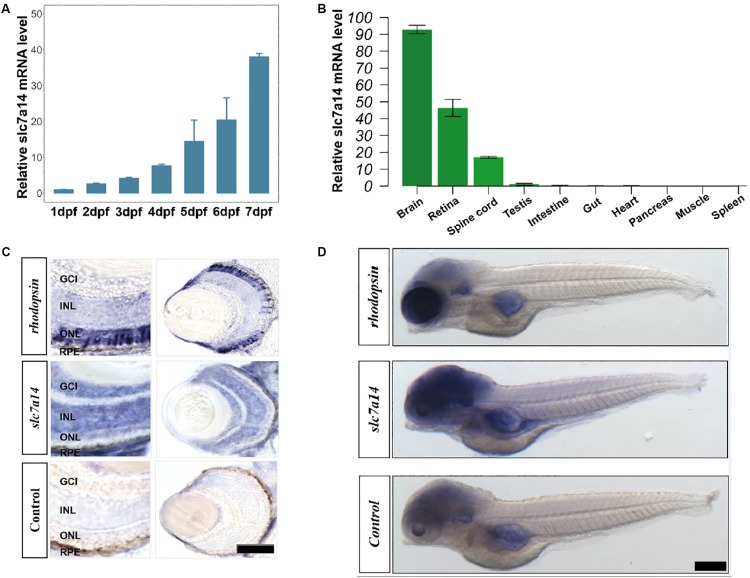
Spatiotemporal expression pattern of *slc7a14* in zebrafish. **(A)** The *slc7a14* expression increased remarkably from 1 to 7 dpf. **(B)** Spatial expression pattern of *slc7a14* in 3-month-old adult male zebrafish. **(C)** *In situ* hybridization (ISH) of *slc7a14* in retinas and eyes. ONL, outer nuclear layer; INL, inner nuclear layer; GCL, ganglion cell layer; RPE, retinal pigment epithelium. Scale bar = 50 μm. **(D)** ISH of *slc7a14* in embryos. Scale bar = 100 μm.

Adult zebrafish were used for tissue-specific qRT-PCR. Zebrafish embryos (*n* = 20 for each PCR sample) at different time points from 1 day post fertilization (dpf) to 7 dpf were harvested for time series qRT-PCR. Total RNA was extracted with Trizol, and the cDNA products were used for qRT-PCR with SYBR green (Roche Applied Science, Germany). For qRT-PCR, each replicate was run in triplicate. The relative gene expression was quantified with a StepOnePlus Real-time PCR System (Life Technologies, United States). qRT-PCR experiments for Figures 1A,B were biologically repeated three times.

### *In situ* Hybridization

Embryos for *in situ* hybridization (ISH) of *slc7a14* were collected at 5 dpf as previously described (Hensley et al., 2011; Zhang et al., 2013). *Slc7a14-*specific DNA fragments 584 bp in length were amplified from the zebrafish cDNA library and cloned into the pGEM-T Easy vector (Promega, United States). The PCR primers were as follows: forward, 5′-CAGCACATACCAGCGATACG-3′, and reverse, 5′-CGATGAATCGCTTCCTCAT-3′. Riboprobes against *slc7a14* mRNA were generated, and the *slc7a14* sense probe was used as a negative control. A rhodopsin antisense probe was used as a positive control. To maximize comparability between groups, all embryos were treated and stained at the same time. The hybridization, washing and destaining steps were performed following protocols described previously (Hensley et al., 2011; Zhang et al., 2013).

### Morpholino Knockdown Experiments

Morpholino oligonucleotide was designed to target the splice site of the *slc7a14* gene, which spans the first intron and the coding region of the second exon (5′-CAAGGCTGAGGACAGAATAAGATGA-3′). Our previous study has verified the splice modifying effect of this MO at 3 dpf (Jin et al., 2014). Moreover, a rescue experiment using a mutated *slc7a14* mRNA has provided further evidence in elucidating the specificity of this MO (Supplementary Figure S5). The standard control MO sequence was as follows: 5′-CCTCTTACCTCAGTTACAATTTATA-3′. Both MOs were synthesized by Gene-Tools, LLC (Corvallis, OR, United States). The *slc7a14* targeting MO was microinjected into the yolks of one-cell stage embryos with three different doses (2.0, 4.0, and 6.0 ng) (Leung et al., 2008; Rosen et al., 2009). Morphological analyses were performed at 3 or 5 dpf (Huang et al., 2018; Ouyang et al., 2019).

### Ocular Measurement and Morphological Analysis

Measurement of eyeball size was performed as previously described (Li et al., 2012; Huang et al., 2018). For data collection, 8 to 15 larvae were included in each trial, and the trials were performed in triplicate. Images of the lateral and vertical view of each larva were captured by a microscopic camera (SZX116, OLYMPUS, Japan). Body length, eye area, axial length, optic tectum size, inter-eye distance and brain size were calculated by the built-in program (OLYMPUS cellsens standard 1.14).

### *Slc7a14* mRNA Preparation and Rescue Experiments

To rescue the knockdown phenotype of *slc7a14*-deficient morphants, the full-length zebrafish *slc7a14* cDNA and human *SLC7A14* cDNA along with the T7 promoter sequence and Kozak sequence (at the 5′ end) were cloned in the pUC57 vector, respectively. The DNA templates were both synthesized by Sangon Biotech (Sangon, China) directly. The amplification primers containing T7 promoter for zebrafish *slc7a14* cDNA were as follows: 5′-TAATACGACTCACTATAGGGGCCACCATGAGCGGCCTCT TCGCC-3′ and 5′- CTCATTCCAAAGGATCATCCAGATCGTC GTCGACCACCAG-3′. The amplification primers containing T7 promoter for human *SLC7A14* cDNA were as follows: 5′-TAATACGACTCACTATAGGGGCCAC-3′ and 5′- CTACTC TGGAGAGTAATCTAACTCATC-3′. To provide further validation of the MO, the mutated zebrafish *slc7a14* cDNA with a nonsense mutation (c.142A > T) was synthesized on the basis of the full-length zebrafish *slc7a14* cDNA by Sangon Biotech. The amplification primers for the mutated full-length zebrafish *slc7a14* cDNA were the same as that of the full-length zebrafish *slc7a14* cDNA. High-fidelity PCR template DNA was purified using a QIAquick PCR Purification Kit (Qiagen, Germany). Capped full-length mRNAs were synthesized with an mMESSAGE mMACHINE^TM^ T7 ULTRA Transcription Kit (Invitrogen, United States) and then purified with an RNeasy Mini Kit (Qiagen) following the manufacturer’s instructions. Subsequently, full-length zebrafish *slc7a14* mRNAs, or mutated full-length zebrafish *slc7a14* mRNAs, or human *SLC7A14* mRNAs was co-injected with a targeting MO into one-cell stage embryos at a final concentration of 200 ng/μL (Yuan and Sun, 2009).

### Visual Motor Response and Optokinetic Response Assay

Visual motor response (VMR) analysis was conducted following standard procedures at 5 dpf (Emran et al., 2008; Liu et al., 2017). For data collection, each experimental group included 12 larvae in a 96-well plate, and 3 h of dark adaption was allowed before the behavior tests. A ZebraBox (VMR machine; ViewPoint 2.0, France) was set to give 3 rounds of ON and OFF light stimuli (30 min for each stimulus) to the larvae. The larval activity during the 150 s spanning the time of light switching was recorded. Optokinetic response (OKR) analysis was performed according to previous protocols at 5 dpf (Rinner et al., 2005; Brockerhoff, 2006; Huang and Neuhauss, 2008). OKR software (ViewPoint OKR 2.0, France) was used to plot the eye movements of the larvae over 1 min. All behavioral analyses were performed at 5 dpf.

### Immunohistochemistry

Zebrafish morphants were fixed in 4% paraformaldehyde at 5 dpf and gradually dehydrated with 15, 22.5, and 30% sucrose. Subsequently, 20 μm-thick frozen sections were collected and stained with anti-zpr-1 (mouse, 1:500; abcam, United Kingdom), anti-zpr-2 and anti-zpr-3 zebrafish-specific antibodies (mouse, 1:400; Zebrafish International Resource Center, United States), respectively at 4°C overnight. The sections were incubated with donkey anti-mouse IgG secondary antibodies conjugated with Alexa Fluor 594 (1:200) for 2 h at room temperature. DAPI (4,6-diamidino-2-phenylindole) was used for nuclear staining. After the coverslips were mounted, images were captured with a confocal microscope (TCS SP8, Leica, Germany).

### TUNEL Assay

For a terminal deoxynucleotidyl transferase (dUTP) nick-end labeling (TUNEL) assay, we followed standard protocols using a One-Step TUNEL Assay Kit (Beyotime, China). Briefly, ocular tissues were fixed with 4% paraformaldehyde, rinsed with PBS, and permeabilized with 0.5% Triton X-100 to obtain the fragmented DNA of apoptotic cells. Then, TUNEL detection solution was added to each sample, and the samples were incubated for 60 min at 37°C in the dark. Finally, the sections were rinsed with PBS again and costained with DAPI (4,6-diamidino-2-phenylindole).

### Quantification and Statistical Analysis

To quantify the relative expression levels of zpr-1, zpr-2, and zpr3, we wrote an R program to count the normalized fluorescence intensities in the zebrafish retinas. Specifically, the fluorescence intensities of each image were normalized by the areas of ONL (The ONL areas were drew and defined manually). To quantify the number of apoptotic retinal cells (including RPE cells) at 3 and 5 dpf, the numbers of TUNEL + cells were manually counted. The statistical analyses were performed using GraphPad Prism 5, SPSS software (version 20) and R software (version 3.5.3). For comparison between the two groups, unpaired Student’s *t*-test was applied. For multiple comparisons, one-way Analysis of Variance (ANOVA) followed by Tukey’s *post hoc* test or Games–Howell test was applied. Bar plots were shown as the mean ± s.e.m. Statistical significance was defined as a *P*-value less than 0.05. ^∗^*P* < 0.05, ^∗∗^*P* < 0.01, ^∗∗∗^*P* < 0.001.

## Results

### *Slc7a14* Is Highly Expressed in the Eye, Brain, and Spinal Cord in Zebrafish

*Slc7a14* is highly expressed in mammalian retinas (Jin et al., 2014). However, whether the spatiotemporal expression patterns of *slc7a14* are conserved among different species has remained unclear. To answer this question, we tested *slc7a14* expression during early embryogenesis in zebrafish. qRT-PCR showed that *slc7a14* expression increased markedly from 1 to 7 dpf (Figure 1A). Next, we analyzed the expression levels in different tissues and found that *slc7a14* was exclusively expressed in neural tissues, including the brain, spinal cord and retina (Figure 1B). To further clarify the localization of *slc7a14* in zebrafish, we conducted ISH experiments. Consistent with the qRT-PCR results, the ISH results revealed that *slc7a14* was highly expressed in the brains and eyes of zebrafish compared to other tissues (Figure 1D). In the eye, *slc7a14* was highly expressed in the outer nuclear layer (ONL), inner nuclear layer (INL), ganglion cell layer (GCL), and retinal pigment epithelium (RPE) layer (Figure 1C). These results demonstrate that *slc7a14* is exclusively expressed in both the neural retina and RPE, implicating its important role in the retina.

### Knockdown of *slc7a14* Led to Marked Ocular Changes in Zebrafish

**FIGURE 2:**
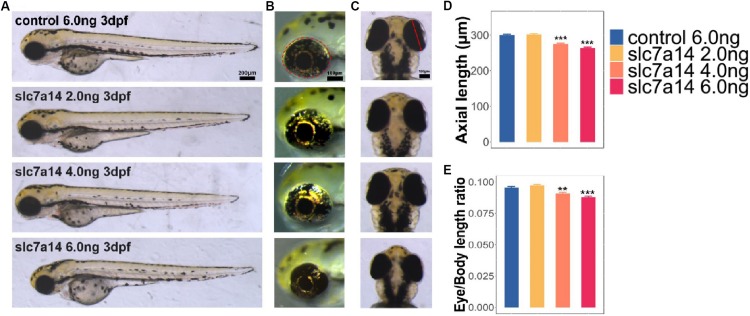
Morphology of *slc7a14*-deficient zebrafish morphants. **(A)** Lateral view of zebrafish larvae. Embryos injected with doses of 4.0 and 6.0 ng MOs exhibited apparent microphthalmia. Scale bar = 200 μm. **(B)** Magnified view of larval eyeballs showing the significant reductions in eye area with high-dose MOs. Scale bar = 100 μm. **(C)** Vertical view of larval eyeballs showing the reductions in eye axis length with high-dose MOs. Scale bar = 100 μm. **(D,E)** Quantification of eye axis length and the axis-to-body length ratio. *N* = 46 in each group. Bar plots are shown as the mean ± s.e.m. Data was analyzed using One-way ANOVA followed by Tukey’s *post hoc* test, ^∗∗^*P* < 0.01, ^∗∗∗^*P* < 0.001 significantly different from control 6.0 ng group.

To test the function of the *slc7a14* gene in zebrafish eyes, we used MO that targets the splice sites of the *slc7a14* gene to construct a knockdown model (Jin et al., 2014). Three different concentrations of *slc7a14* MOs, together with a standard control MO, were microinjected into the yolks of embryos, respectively. Strikingly, both the high-concentration knockdown groups (injected with 4.0 and 6.0 ng) exhibited apparent microphthalmia phenotypes, including a shortened eye axis, reduced eye area and a decreased eye axis length-to-body length ratio (Figures 2A–C). Compared to zebrafish received control MO, zebrafish that received 6.0 ng *slc7a14*-MO showed a 12.26% reduction in eye axis length, a 20.17% reduction in eyeball size and a 7.87% reduction in the eye axis length-to-body length ratio (Figures 2D,E). Taken together, our results demonstrated that temporary silencing of the *slc7a14* gene led to significant pathological ocular phenotypes in zebrafish.

### Aberrant Rod Photoreceptors and Peripheral RPE in *slc7a14*-Deficient Morphants

**FIGURE 3:**
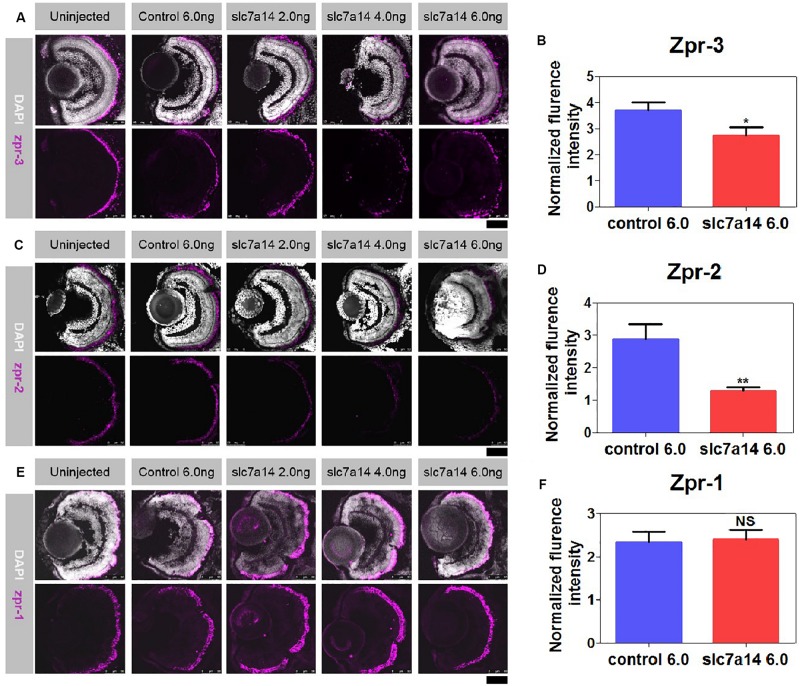
Immunostaining of zpr-1, zpr-2, and zpr-3 in *slc7a14*-deficient morphants. **(A)** In the peripheral retina, the high-dose *slc7a14*-MOs led to sharp reductions in zpr-3. Weaker fluorescence signals were detected in the high-MO dose groups (*slc7a14* 4.0 ng and *slc7a14* 6.0 ng) than in the control group. **(B)** Statistical results for zpr-3 (*n* = 10 for each group). **(C)** The high-dose *slc7a14*-MOs led to significant reductions in zpr-2. There were few fluorescence signals in the peripheral retina in the high-MO dose group (*slc7a14* 6.0 ng). The RPE in the low-MO dose group (*slc7a14* 2.0 ng) was relatively normal compared to that in the wild-type group and control group. **(D)** Statistical results for zpr-2 (*n* = 10 for each group). **(E)** No significant changes were found in cone photoreceptors. **(F)** Statistical results for zpr-1 (*n* = 5 for each group). Scale bar = 50 μm. Bar plots were shown as the mean ± s.e.m. *T*-test was performed between the two groups. ^∗^*P* < 0.05, ^∗∗^*P* < 0.01.

To further determine the retinal structural changes of *slc7a14*-knockdown morphants, we used the specific markers zpr-1, zpr-2, and zpr-3 to observe cones, the RPE and rods, respectively. Compared to zebrafish received control MO, zebrafish that received 4.0 and 6.0 ng *slc7a14*-MO displayed significant reduction of zpr-3 and in zpr-2 in the retina, while zpr-1 was not altered (Figures 3A–F). Notably, the zpr-2 signals of the 6.0 ng *slc7a14*-MO group decreased to 44.48% of the control group (Figure 3D). Consistently, in the Tg(gad1b:mCherry) zebrafish with *slc7a14* deficiency compared to control, the mCherry signal in the peripheral RPE layer was significantly decreased (Supplementary Figure S1). Altogether, these results suggested that knocking down *slc7a14* led to aberrant rod photoreceptors and peripheral RPE in zebrafish.

### Increased Apoptotic Cells in *slc7a14* Knockdown Retinas

**FIGURE 4:**
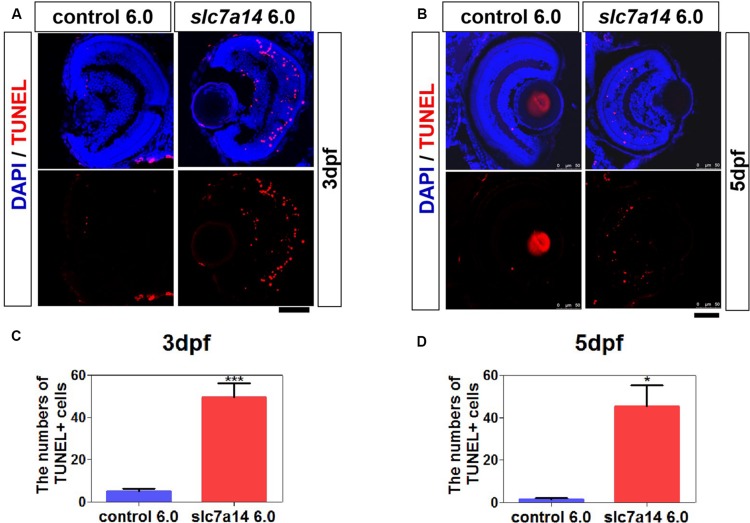
Knockdown of *slc7a14* led to increased apoptosis in the zebrafish retina. TUNEL assay was used to detect apoptosis in larval retinas at 3 and 5 dpf. **(A,B)** *Slc7a14* knockdown resulted in significant increase of TUNEL + cells in the retina including GCL, INL, ONL and RPE at 3 dpf **(A)** and 5 dpf **(B)**. Scale bars = 50 μm. **(C,D)** Statistical results for the numbers of TUNEL positive cell at 3 dpf (*n* = 6 for each group) and at 5 dpf (*n* = 3 for each group). Bar plots were shown as the mean ± s.e.m. *T*-test was performed between the two groups. ^∗^*P* < 0.05, ^∗∗∗^*P* < 0.001.

To determine whether *slc7a14* knockdown leads to cell death in the retina, we performed TUNEL staining. In morphants with standard MOs (6.0 ng), apoptotic signals were barely detected at 3 and 5 dpf (Figures 4A,B). However, the number of apoptotic cells was significantly increased in the retina and the RPE of *slc7a14*-MO 6.0 ng group compared to the control group (Figures 4A–D). Notably, we noticed that the TUNEL signals appeared in the INL, ONL and RPE layer (Figures 4A,B). These results suggested that knockdown of *slc7a14* in zebrafish increased retinal cell apoptosis.

### Impaired Visual Behaviors of *slc7a14*-Knockdown Zebrafish

**FIGURE 5:**
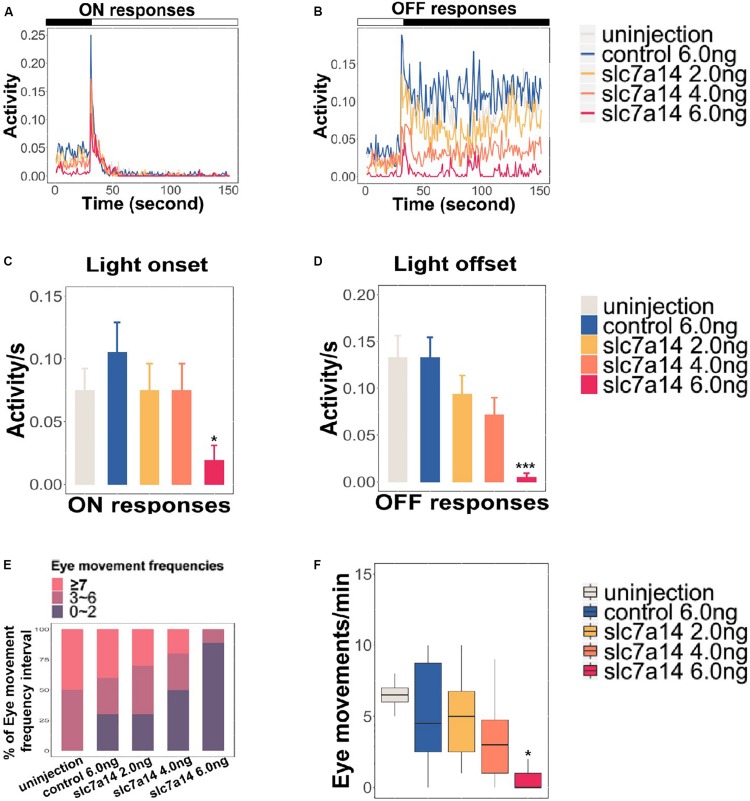
*Slc7a14*-deficient zebrafish morphants showed defective visual behaviors. **(A,B)** VMR testing in *slc7a14*-deficient zebrafish morphants. Larvae injected with *slc7a14*-MO (6.0 ng) showed a weaker ON response and a significantly attenuated OFF response compared to control larvae. *N* = 12 in each group. **(C,D)** Quantification of the VMR. *N* = 12 in each group. **(C)** Larvae injected with *slc7a14* MO (6.0 ng) showed a reduced ON response compared with control larvae. **(D)** Compared with control larvae, larvae injected with 4.0 ng MO and 6.0 ng *slc7a14*-MOs exhibited markedly reduced OFF responses. **(E,F)** OKR testing demonstrated significant reductions in eye movement in the *slc7a14*-deficient zebrafish morphants compared to the control zebrafish. *N* = 10 in each group. VMR testing and OKR testing were repeated three times, respectively. Data was analyzed using One-way ANOVA followed by Games–Howell test, ^∗^*P* ≤ 0.05, ^∗∗∗^*P* < 0.001 significantly different from control 6.0 ng group.

To further determine the effect of *slc7a14* knockdown on visual function in zebrafish, we tested the VMR and OKR to evaluate visual behavior. Based on a previously described protocol (Jin et al., 2014), we applied three ON and three OFF light stimuli to 5 dpf larvae in 96-well plates. Morphants injected with 6.0 ng of *slc7a14*-MO displayed reduced activity in both the ON (0.019) and OFF (0.006) conditions (Figures 5A–D). Of note, the OFF responses appeared to be more significantly impaired than the ON responses in *slc7a14*-deficient morphants. Similarly, the *slc7a14*-deficient zebrafish showed impaired OKR. The eye movements were markedly decreased in *slc7a14*-deficient zebrafish compared to control zebrafish (Figures 5E,F). Overall, these findings demonstrated that *slc7a14* deficiency led to severe visual behavioral impairments in zebrafish.

### Reversibility of the Knockdown Effects With mRNA Compensation

**FIGURE 6:**
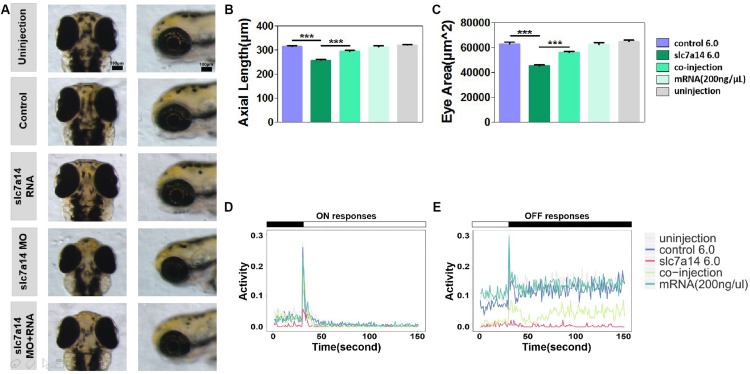
*Slc7a14* mRNA compensation rescued the phenotypes in *slc7a14*-deficient morphants. **(A)** Magnified vertical and lateral views of larval eyeballs. Larvae injected with *slc7a14* MO and full-length *slc7a14* mRNA showed normal-sized eyeballs at 3 dpf. Scale bar = 100 μm. **(B,C)** Statistical results for the axial length and ocular area. *N* = 30 in each group. **(D,E)** VMR testing showed dramatic recovery of both the ON and OFF responses in *slc7a14*-deficient zebrafish compensated with mRNA compared to *slc7a14*-deficient zebrafish without compensation. *N* = 12 in each group. Rescue experiments were repeated three times. Bar plots are shown as the mean ± s.e.m. Data was analyzed using One-way ANOVA followed by Tukey’s *post hoc* test, ^∗∗∗^*P* < 0.001 significantly different from *slc7a14* 6.0 ng group.

To further verify that the ocular phenotypes found in *slc7a14*-deficient morphants were not due to off-target effects, we performed a rescue experiment with mRNA compensation. Full-length *slc7a14* mRNA and *slc7a14* MO (6.0 ng) were coinjected into embryos. We examined the phenotypes by measuring ocular size and VMR. Interestingly, dramatic recovery from microphthalmia was observed in the mRNA- and MO-coinjected larvae (Figures 6A–C). In addition, mRNA- and MO-coinjected larvae showed significantly rescued ON and OFF responses (Figure 6D). To confirm whether the biological function of *slc7a14* was conserved between human and zebrafish, we performed another rescue experiment using human full-length *slc7a14* mRNA. As expected, consistent rescue effects were observed in the zebrafish (Supplementary Figure S2). Overall, we found that the phenotypes of *slc7a14* knockdown were reversible upon compensation with full-length mRNA in the *slc7a14-*deficient morphants.

## Discussion

We have reported *SLC7A14* as a novel disease-causing gene of autosomal recessive RP (Jin et al., 2014). In this study, we further demonstrated in zebrafish that knocking down *slc7a14* resulted in significant ocular size defects, retinal cell apoptosis and visual behavioral impairment. Importantly, the disease phenotypes caused by *slc7a14* deficiency were ameliorated by mRNA compensation.

A previous study revealed the spatiotemporal expression pattern and the developmental expression trend of *slc7a14* in the retinas of mice (Jin et al., 2014). In this study, ISH confirmed the existence of the same spatiotemporal pattern in zebrafish. We also found that this gene is abundantly expressed in the GCL, INL, and ONL of the zebrafish retina, consistent with previous findings in mice (Jin et al., 2014). In addition, we discovered, for the first time, that *slc7a14* is also expressed in RPE cells. Furthermore, retinal immunohistochemistry demonstrated that the RPE-specific zpr-2 signal was significantly reduced in *slc7a14*-knockdown larvae compared to control larvae, collectively suggesting a key role of *slc7a14* in RPE.

Our VMR results demonstrated that *slc7a14* deficiency lead to visual behavioral impairments in zebrafish at 5 dpf (Figure 5). The VMR is a vision based movement responding to light ON and OFF. Considering that *slc7a14* was possibly knocked down in brain and spinal cord as well, the results of the OKR and VMR assays may be due to other neurological effects. We then carried out additional morphological analysis to address this issue. We measured the brain size, the inter-eye distance and the size of optic tectum. Although the absolute values of the brain size and the inter-eye distance were much smaller in the MO group (Supplementary Figures S3A,B), there was no significant difference of the inter-eye distance/brain size ratio between the MO and control groups at 3 dpf (Supplementary Figure S3C). Notably, no significant differences were observed at 5 dpf (Supplementary Figures S3D–F). Moreover, knockdown of *slc7a14* showed no significant changes in the size of optic tectum (Supplementary Figure S4). Additionally, we did not observe any curved body morphology in MO groups. Finally, it is noteworthy that *slc7a14* knockdown mainly affect the VMR OFF response rather than ON response, which cannot be explained by motor deficits (Figures 5A–D and Supplementary Figures S2C,D). Furthermore, the expression of zpr3 (rod specific) decreased more significantly than that of zpr1 (cone specific), indicating a more severe influence in rods in *slc7a14* deficient larvae (Figure 3). Conversely, we found that in a cone-specific pde6c mutant fish line (Zhang et al., 2016), the ON response was affected more severely than the OFF one. This finding implied that cone- and rod-specific mutants may show different performances in ON and OFF responses. Based on the previous and current findings, we assume the defective VMR responses were mainly caused by visual defects. Since we have not checked whether MO continued to be effective beyond 3 dpf, the defects observed at 5 dpf may be due to reduced *slc7a14* transcript abundance through 3 dpf only.

To avoid false positive results in the retinal immunohistological analysis, we used a Tg(gad1b:mCherry) zebrafish line for the knockdown experiments. Gad1b (glutamate acid decarboxylase 1 beta) is known to catalyze the conversion of L-glutamic acid into γ-aminobutyric acid (GABA) (Legay et al., 1986; Straub et al., 2007; Zhang et al., 2010). The mCherry signals in this transgenic zebrafish line indicate the expression of gad1b in RPE and amacrine cells. We confirmed dramatic declines in the mCherry signals in both RPE and amacrine cells in *slc7a14*-deficient Tg(gad1b:mCherry) larvae compared to control larvae, implying a possible role for *slc7a14* in RPE/amacrine development or maintenance (Supplementary Figure S1). Previously, several studies showed that the inhibitory signaling progresses laterally through the retina. It was mediated mainly by GABA via amacrine cells and horizontal cells (Schur et al., 2018; Moore-Dotson and Eggers, 2019). The mCherry signal in amacrine cells was weakened, implicating that the inhibition signal may be interrupted. Several studies showed that valproic acid, a clinically used anticonvulsant, could increase GABAergic signaling in retina and had entered the clinical trial stage for treating of RP (Mitton et al., 2014).

In summary, we revealed the spatiotemporal patterns and retinal expression patterns of *slc7a14* in zebrafish and found that knockdown of *slc7a14* led to significant pathological ocular phenotypes as well as visual impairment. These findings provide insight into the biological role of *SLC7A14* in the human retina and support the development of therapeutic strategies for *SLC7A14*-related retinal degenerative diseases.

## Data Availability Statement

The datasets generated for this study are available on request to the corresponding author.

## Ethics Statement

The animal study was reviewed and approved by The Eye Hospital of Wenzhou Medical University.

## Author Contributions

Z-BJ conceived and supervised the study. Z-BJ and LX provided funding supports. Y-YZ, LX, R-JS, NZ, and X-RW carried out the experiments. Y-YZ and X-RW performed the data analysis. Y-YZ, LX, R-YH, and S-SZ wrote the manuscript. Z-BJ, FL, and JQ revised the manuscript.

## Conflict of Interest

The authors declare that the research was conducted in the absence of any commercial or financial relationships that could be construed as a potential conflict of interest.

## Footnotes

1 http://www.sph.uth.tmc.edu/RetNet/

2 http://retinogenetics.org/
