# Supplementary material for: Slc7a14 Is Indispensable in Zebrafish Retinas
Source: Front Cell Dev Biol. 2019 Dec 12;7:333. doi: 10.3389/fcell.2019.00333 (PMC6920099; doi:10.3389/fcell.2019.00333)
Supplement: Supplementary file 1 [file Table_1.pdf]

Supplementary Material

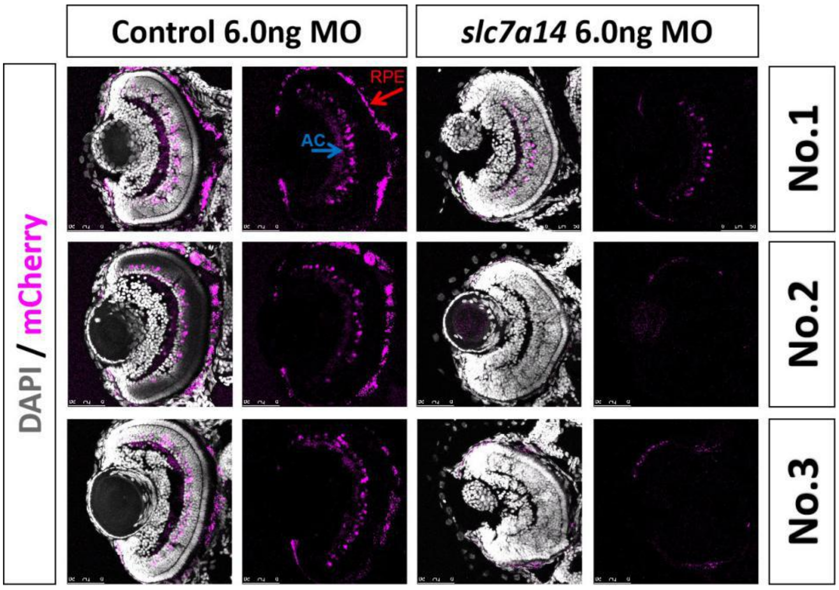

Fig. S1 Knockdown of *slc7a14* in Tg(gad1b:mCherry) transgenic zebrafish.

The mCherry fluorescent signals occurred specifically in amacrine cells (ACs) and RPE cells in Tg(gad1b:mCherry) larval retinas at 3 dpf. Injection of *slc7a14* MO at 6.0 ng led to dramatic declines in mCherry signals in both amacrine cells and RPE cells. Scale bars = 50 μm.

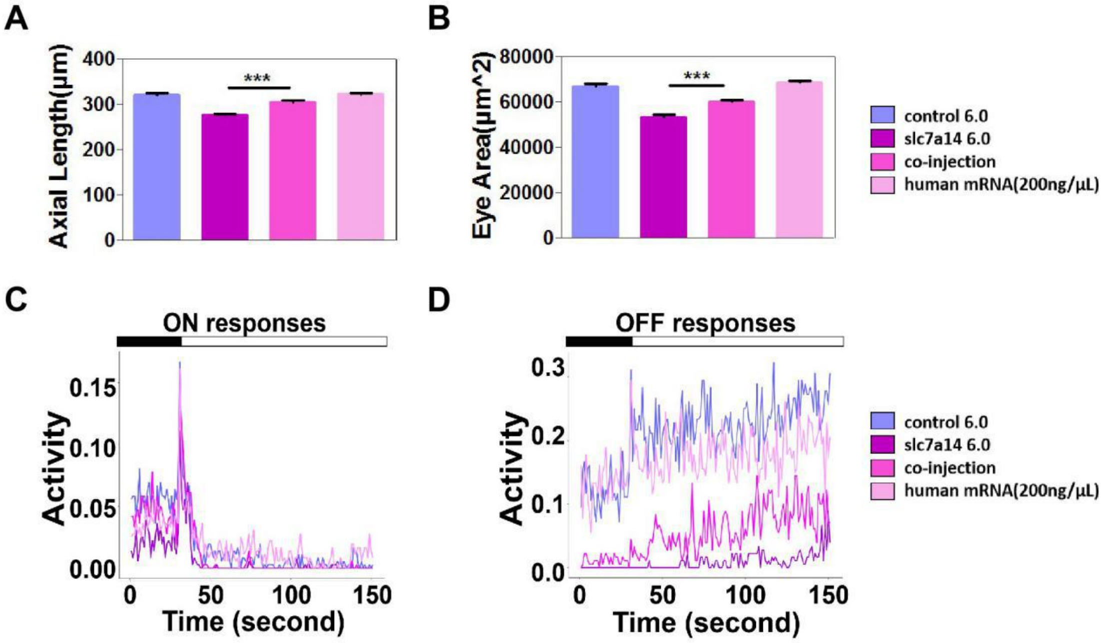

Fig. S2 Eye size measurements and VMR responses in the rescue experiments using human *SLC7A14* mRNA.

(A, B) Statistical results for the axial length and ocular area (*N* = 35 in each group). Larvae injected with *slc7a14* MO and human full-length *SLC7A14* mRNA showed relatively normal sized eyeballs at 3 dpf. Bar plots were shown as the mean±s.e.m. Data was analysed using One-way ANOVA followed by Tukey's post-hoc test, $***P < 0.001$ significantly different from *slc7a14* 6.0 ng group. **(C, D)** VMR testing showed rescue effects of both the ON and OFF responses in *slc7a14*-deficient zebrafish when compensating with human mRNA. *N* = 12 in each group. Rescue experiments were repeated three times. These results were consistent with the observations in the zebrafish *slc7a14* mRNA compensation experiments.

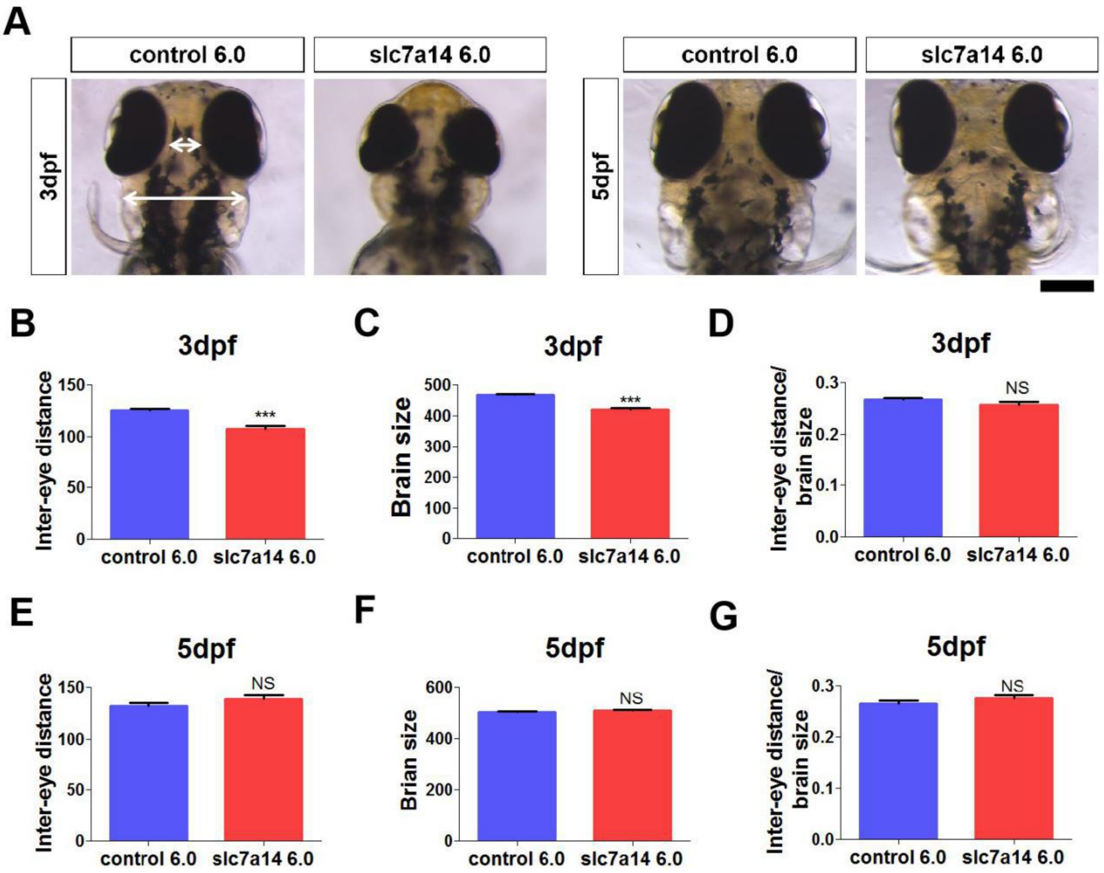

Fig. S3 Measurements of the inter-eye distance and brain size in *slc7a14* knockdown larvae at 3dpf and 5dpf.

(A) Vertical view of larvae injected with *slc7a14* MO and control MO at 3dpf and 5 dpf. The very left image showed the inter-eye distance (short double-ar row) and brain size (long double-arrow) we calculated. Scale bar = 200 μm. **(B-G)** Statistical results of larvae for the inter-eye distance, brain size and inter -eye distance/brain size ratio at 3 dpf **(B-D)** and 5 dpf **(E-G)**. *N* = 19 for eac h group. The experiments were repeated three times. Bar plots were shown as the mean±s.e.m. T-test was performed between the two groups. $***P < 0.001$. NS: nonsignificant.

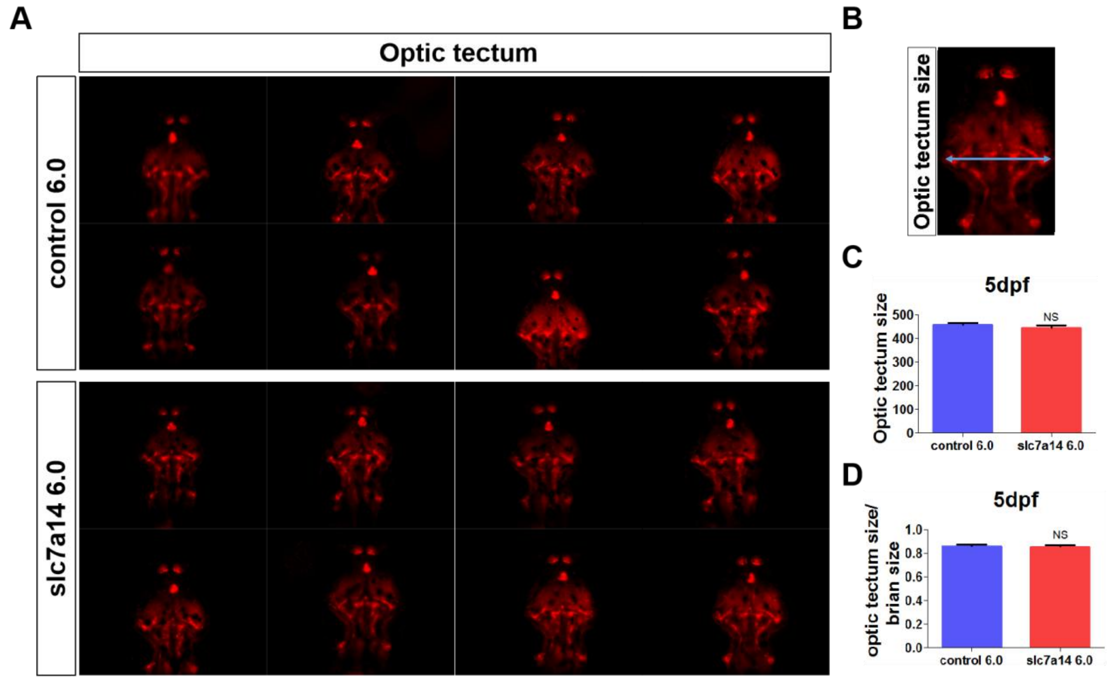

Fig. S4 Knockdown of *slc7a14* had no significant changes in optic tectum
(A) Vertical view of the optic tectum in the Tg(gad1b:mCherry) transgenic larvae with the injections of *slc7a14* MO and control MO at 5 dpf. **(B)** Measurements of the size of optic tectum. **(C, D)** Statistical results for the optic tectum size and optic tectum size/brain size ratio at 5dpf. $N = 8$ in each group. Bar plots were shown as the mean±s.e.m. T-test was performed between two groups. NS: no significance.

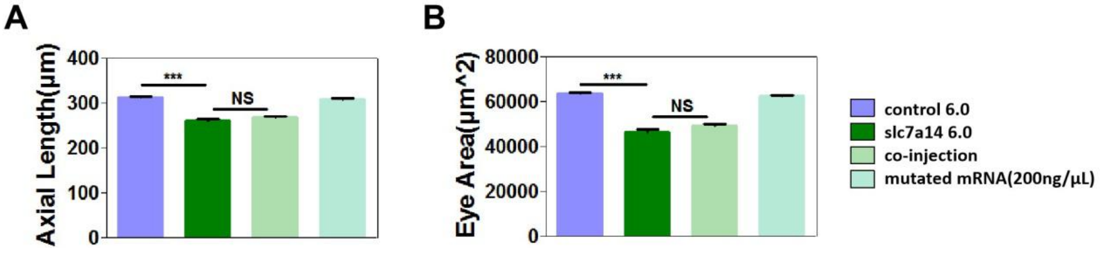

Fig. S5 Mutated zebrafish *slc7a14* mRNA did not rescue the phenotype of the eye size in *slc7a14*-deficient morphants.

(A, B) Statistical analyses of the axial length and the ocular area (N = 20 in each group). Unlike the observation we got in figure 6, we could not observe a significant rescue effect using the mutated *slc7a14* zebrafish mRNA in *slc7a14*-deficient morphants at 3 dpf. The experiments were repeated three times. Data was analysed using One-way ANOVA followed by Tukey's post-hoc test, ***$P < 0.001$ significantly different from *slc7a14* 6.0 ng group. NS: not significantly different from *slc7a14* 6.0 ng group.
